# Glutamatergic mechanisms in early salience processing

**DOI:** 10.3389/fphar.2025.1601797

**Published:** 2025-07-01

**Authors:** Denise Elfriede Liesa Lockhofen, Nils Hübner, Ranjan Debnath, Karl Philipp Rumpf, Michael Sander, Matthias Wolff, Daniel Luxi, Lukas Roller, Christoph Mulert

**Affiliations:** ^1^ Centre of Psychiatry, Justus-Liebig University, Giessen, Hessen, Germany; ^2^ Department of Anaesthesiology, Intensive Care Medicine and Pain Therapy, University Hospital of Giessen, UKGM, Justus-Liebig University Giessen, Giessen, Germany

**Keywords:** schizophrenia, glutamate, glutamate hypothesis, early salience, attention, ketamine, additional singleton task, EEG

## Abstract

**Introduction:**

Patients with schizophrenia frequently experience inadequate attribution of motivational salience, possibly related to impaired attentional processing and dysfunctional reward learning. According to the “glutamate hypothesis of schizophrenia”, glutamatergic dysregulations can contribute to the emergence of psychotic symptoms and cognitive deficits in patients with schizophrenia. Blocking the N-methyl-D-aspartate receptor (NMDAR) with NMDAR antagonists such as ketamine can lead to temporary schizophrenia-like symptoms in healthy volunteers, including cognitive and attentional impairments.

**Method:**

The present study investigated how the administration of a subclinical dose of ketamine compared to placebo affects the interaction of attention and reward. 27 healthy volunteers received either an intravenous infusion of ketamine or a placebo. Subsequently, an EEG was recorded while the subjects performed a visual attention task with salient, reward-related distractors.

**Results:**

The results demonstrate that ketamine primarily interfered with distractor processing, with little to no effect on target or reward processing. In addition, ketamine administration led to an increase in gamma band power compared to placebo and in salient distractor trials compared to target-only trials. Interestingly, these effects were related to the occurrence of negative symptoms.

**Discussion:**

Therefore, the present findings further emphasize the role of the glutamate system in the development of dysfunctional gamma band oscillations, early salience processing alterations and negative symptoms in patients with schizophrenia.

## 1 Theory

Selective attention controls the way we direct our awareness to relevant stimuli in our environment, while at the same time ignoring irrelevant information. Recent studies have shown that attentional selection is not just determined by salience-driven bottom-up processes and goal-directed top-down processes, but can also be influenced by reward (Awh et al., 2012). Monetary rewards, for example, can enhance or impair attentional selection, depending on the rewarded stimulus (Della Libera and Chelazzi, 2006; Engelmann et al., 2009; Anderson et al., 2013; Bourgeois et al., 2017; Chela et al., 2013; Hickey et al., 2010; Le Pelley et al., 2015; Watson et al., 2019; Lockhofen et al., 2021).

This interaction between attention and reward might be particularly important when it comes to psychiatric disorders such as schizophrenia. Patients suffering from schizophrenia typically show a wide range of cognitive impairments across several domains, including attention, processing speed, working memory, verbal learning, memory and executive functioning (McCutcheon et al., 2023; Bowie and Harvey, 2006; Nuechterlein et al., 2004). Attentional deficits, in particular, are considered to be a hallmark of schizophrenia (Lesh et al., 2011). Schizophrenic patients display significant impairments in visual attention (Caprile et al., 2015; John et al., 2018; Hahn et al., 2012) and attentional control (Luck and Gold, 2008). Furthermore, they experience difficulties discriminating between relevant and irrelevant information by focusing on non-predictive cues (Morris et al., 2013) or distractors (Gur et al., 2007), which might be related to a tendency of schizophrenic patients to extensively focus (hyperfocus) on irrelevant information (Luck et al., 2023).

In addition to attentional impairments, patients with schizophrenia also show motivational impairments (Murray et al., 2008), which seem to play an important role in the genesis of positive (Kapur, 2003) and negative (Barch and Dowd, 2010; Gold et al., 2008; Leroy et al., 2020) symptoms. In particular, the “aberrant salience model” proposes that the development of psychotic symptoms, such as delusions, is driven by the inappropriate attribution of motivational salience to irrelevant stimuli, which in turn causes them to attract attention (Kapur, 2003; Roiser et al., 2013). It has been assumed that this aberrant attribution of motivational salience results from abnormal dopamine signaling and functional alterations in the striatum and the hippocampus (Roiser et al., 2013; Corlett et al., 2009; Lodge and Grace, 2007; Winton-Brown et al., 2014). However, recent studies have suggested that the aberrant salience phenomenon and its underlying dopaminergic dysregulations could also be secondary consequences of other brain disturbances, such as dysfunctions in glutamatergic signaling (McCutcheon et al., 2021; Panayi et al., 2023).

The “glutamate hypothesis of schizophrenia” (Javitt and Zukin, 1991) is based on the early observations that N-Methyl-D-aspartate-receptor (NMDAR) antagonists, such as phencyclidine (PCP) and ketamine, produce schizophrenia-like symptoms in healthy individuals and can intensify or reexacerbate psychotic symptoms and cognitive deficits in patients with schizophrenia (LUBY et al., 1959; Rosenbaum et al., 1959; Olney and Farber, 1995; Krystal et al., 2003). These findings led to the proposal that schizophrenia might be the result of an NMDAR hypofunction (Olney, 1989). The so-called “disinhibition model” suggests that NMDAR hypofunction on fast-spiking γ-aminobutyric acid (GABA)-ergic interneurons leads to a disinhibition of glutamate neurons and an increased release of glutamate, especially in prefrontal regions (Homayoun and Moghaddam, 2010; Lisman et al., 2008; Moghaddam and Javitt, 2012). The idea that abnormalities in NMDAR-mediated neurotransmission are involved in the pathophysiology of schizophrenia is supported by genetic linkage studies (Harrison and Bannerman, 2023; Trubetskoy et al., 2022; Singh et al., 2022) as well as by clinical phase 2 studies, demonstrating that substances which increase synaptic levels of NMDAR co-agonist glycine show a significant effect on negative symptoms and cognitive impairments associated with schizophrenia (Fleischhacker et al., 2021; Umbricht et al., 2014). In line with these findings, it was shown that schizophrenia was associated with elevated levels of glutamatergic metabolites in several brain regions, such as the basal ganglia, the thalamus and the medial temporal lobe (Merritt et al., 2016).

As mentioned above, blocking the NMDAR with NMDA antagonists, such as ketamine, can induce transient schizophrenia-like symptoms in healthy individuals (Beck et al., 2020; Pennybaker et al., 2017). Several studies have found that acute ketamine administration in healthy subjects impairs cognition across all domains (Zhornitsky et al., 2022) and induces attentional deficits similar to those found in schizophrenic patients (Oranje et al., 2000; Umbricht et al., 2002). Ketamine was found to impair early attentional processes (Musso et al., 2011; Watson et al., 2009) as well as later voluntary attentional control (Fuchs et al., 2015) and can also have a detrimental effect on reward processing. For example, it can prevent the attribution of incentive salience (Chow and Beckmann, 2018; Fitzpatrick and Morrow, 2017) and attenuate the ventral striatal response during reward anticipation in the same way as has been observed in schizophrenic patients (Francois et al., 2016).

The cognitive deficits induced by ketamine administration in healthy participants were found to be accompanied by changes in event-related potentials (ERP, (Oranje et al., 2000; Watson et al., 2009; Gunduz-Bruc et al., 2012; Rosburg and Schmidt, 2018)), which correspond to findings in patients with schizophrenia (Jeon and Polich, 2003). Studying ERP also helps identify which stages of information processing are affected by NMDAR blockade. While early ERP components (N100, N200, P200) are related to attentional selection (Ghani et al., 2020) and seem to be mainly dependent on the properties of the stimulus, later components typically reflect more cognitive or endogenous information processing (Schwertner et al., 2018). The most widely studied ERP is the P300 (Sutton et al., 1965; Bashore and van der Molen, 1991), which is usually assessed using variations of the “oddball” paradigm. Several studies have found that ketamine administration attenuates the amplitudes of the target-related P3b and the distractor-related P3a (Watson et al., 2009; Gunduz-Bruc et al., 2012; Mathalon et al., 2014). While some authors (Schwertner et al., 2018) interpreted this effect as an alteration of perceived stimulus salience or stimulus discriminability, others (Rosburg and Schmidt, 2018) proposed a more general encoding deficit, particularly of new episodic information, after ketamine administration. On the contrary, the effect of ketamine on early EEG components, such as P100 or N100, was rather mixed (Rosburg and Schmidt, 2018). Some studies reported stable or increased amplitudes after ketamine administration (see (Schwertner et al., 2018) for an overview) while others reported decreased amplitudes (Watson et al., 2009; Boeijinga et al., 2007; Schmidt et al., 2013).

However, neural communication is not only determined by anatomical connectivity and activity-dependent changes in neural activity, but also by synchronization of neural oscillations (Fries, 2015). Gamma band oscillations (30–100 Hz) in particular have attracted scientific attention, because they were found to accompany various cognitive and psychological processes (Uhlhaas and Singer, 2010; Uhlhaas et al., 2011), including selective attention (Tiitinen et al., 1993; Tallon-Baudry and Bertrand, 1999; Kaiser and Lutzenberger, 2005; Ray et al., 2008; Magazzini and Singh, 2018). In general, it is assumed that gamma band oscillations are primarily involved in bottom-up processing and perception, whereas top-down influences are more related to alpha-beta band synchronization (Bastos et al., 2012; Michalareas et al., 2016). Our own research has demonstrated that patients with schizophrenia show disturbances in gamma band activity (Leicht et al., 2010), which seem to be related to their psychopathology (Andreou et al., 2015; Mulert et al., 2011; Kornmayer et al., 2018). Furthermore, we found that patients with schizophrenia as well as subjects with schizotypal personality disorder show not only disturbances of gamma band oscillations but also alterations in early salience processing (Kornmayer et al., 2018; Kornmayer et al., 2015). On a microcircuit level, gamma band oscillations depend on an interplay between excitatory and inhibitory networks (E/I balance) that modulate neural responsiveness and allow for the formation of transient links between neuron ensembles (Haider and McCormick, 2009; Uhlhaas, 2013; Uhlhaas et al., 2009). Blocking the NMDAR with ketamine was shown to induce disturbances in gamma band oscillations (Carlén et al., 2012; Hudson et al., 2020; Thiebes et al., 2018; Curic et al., 2019). These abnormalities could be found in resting-state (Nottage et al., 2023; Curic et al., 2021; Grent-‘t-Jong et al., 2018) as well as in task-related EEG data (Grent-‘t-Jong et al., 2018; Shaw et al., 2015).

The main aim of the present study was to further improve our understanding of the glutamate hypothesis of schizophrenia by examining the effect of ketamine on the interplay between top-down attention, early salience and reward processing, as well as on the underlying neurophysiological processes. So far, there are no other studies that have investigated this interaction in healthy participants under ketamine or in the schizophrenia spectrum. To disentangle the effect of ketamine on attention and reward, we employed a variant of the Additional Singleton Task (AST, (Theeuwes, 1991)) that we (Lockhofen et al., 2021) and others (Feldmann-Wüstefeld et al., 2016) have used in previous studies. In this version of the task, subjects have to respond to targets, while at the same time ignoring a physically salient distractor whose color is associated with high or low rewards. This task design is particularly well suited to investigate the interaction of attention and reward processes, since rewards can be associated with stimuli that are not task-relevant and have not been task-relevant before. Thus, the effect of reward will not be influenced by any motivational effects (Le Pelley et al., 2015). The AST allowed us to investigate the N2-posterior-contralateral (N2pc), an attention-sensitive event-related potential, elicited at posterior electrodes at post-stimulus latencies of 200–350 ms. Using a systematic lateralization technique (Hickey et al., 2009; Woodman and Luck, 2003) that takes advantage of the contralateral organization of the visual system and allows us to separate target and distractor processing, we were able to calculate three sub-components of the N2pc: distractor negativity (N_D_), distractor positivity (P_D_) and target negativity (N_T_). The N_D_ and P_D_ components are elicited by laterally presented distractors and are supposed to reflect attentional capture by the distractor (N_D_) and active suppression of the distractor (P_D_). The N_T_ component is elicited by laterally presented targets and has been associated with target prioritization (Lockhofen et al., 2021; Feldmann-Wüstefeld et al., 2016). For more information on the lateralization process see (Feldmann-Wüstefeld et al., 2016). Using this design, the authors (Feldmann-Wüstefeld et al., 2016) have found that distractors associated with high rewards were more likely to capture attention than low reward distractors (increased N_D_ component). At the same time, they required more active suppression (increased PD component). In a previous study (Lockhofen et al., 2021), we used the AST to investigate the difference between rewarding targets (TR group) and rewarding distractors (DR group) and found that the N_D_ component was stronger in the DR group than in the TR group. This effect was accompanied by an increase in frontal activation for the DR group and might reflect a greater need for top-down guidance when rewards were associated with task-irrelevant distractors. Furthermore, we found an increased activation in the value-based attention network that showed time-dependent differences, indicating that the neural mechanisms underlying reward processing might be different for task-relevant and task-irrelevant stimuli (Lockhofen et al., 2021).

As described above, acute ketamine administration can induce symptoms and attentional (Oranje et al., 2000; Umbricht et al., 2002) deficits as well as brain changes (Francois et al., 2016) in healthy individuals similar to those experienced by patients with schizophrenia. Therefore, ketamine administration in healthy subjects during a visual attention task with rewarded distractors should lead to impairments in task performance, along with attention-related changes in EEG measures. Specifically, we expect ketamine to induce positive and negative symptoms similar to those in schizophrenic patients and to increase the subject’s focus on task-irrelevant information. This should be reflected in longer response times and higher error rates during the visual attention task. In the EEG we expect to see increased distractor components (N_D_, P_D_) and decreased target components (N_T_) under ketamine compared to placebo. Additionally, we expect ketamine to modulate high frequency oscillations in the gamma band range, which have previously been associated with selective attention (Tiitinen et al., 1993; Tallon-Baudry and Bertrand, 1999; Kaiser and Lutzenberger, 2005; Ray et al., 2008; Magazzini and Singh, 2018), physical salience (Kornmayer et al., 2018; Kornmayer et al., 2015) and reward processing (HajiHosseini et al., 2012). Based on our previous research (Kornmayer et al., 2018), we assume that ketamine leads to an increase in gamma band power, especially for trials with salient distractors compared to trials without a salient distractor. Since ketamine was also reported to influence reward processing (Chow and Beckmann, 2018; Fitzpatrick and Morrow, 2017), these effects could be dependent on the distractor’s reward-value. Generally, we expect distractors associated with high rewards to draw more attention than distractors associated with low rewards and thus lead to longer response times, increased error rates, increased distractor components (N_D_, P_D_), decreased target components (N_T_) and a stronger gamma band response.

## 2 Method

### 2.1 Ethics statement

The study was approved by the Institutional Review Board of the University of Giessen and carried out in accordance with the latest version of the Declaration of Helsinki. Written informed consent was obtained from all participants.

### 2.2 Participants

A total of twenty-seven volunteers (13 male, mean age = 24 years, SD = 2.9, range: 21–30; 14 female, mean age = 24 years, SD = 2.8, range = 21–29) took part in a randomized, single-blind, crossover study. All were right-handed and had normal or corrected-to-normal vision and no color blindness (assessed with the Snellen vision test and the Ishihara Test for Color Deficiency). Exclusion criteria included any history of psychiatric or neurologic disorder; a previous adverse response to ketamine, any medical conditions that affect hepatic, renal or gastrointestinal functions, cardiac abnormalities, hypertension or a history of substance abuse. Prior to the experiment, participants were informed that correct responses would yield them points and that an equivalent amount of these points would be paid after the experiment in Euros. 1,000 points corresponded to a reward of 4.19 EUR. Thus, 100% correct responses would yield the participant a total of 5,808 points (24.39 EUR). All participants gave their informed written consent to participate in the study. Five participants had to be excluded afterwards because of technical difficulties.

### 2.3 Stimuli and apparatus

Task, stimuli and procedure were similar to the distractor reward (DR) condition of a previous study (Lockhofen et al., 2021). The experiment took place in a dimly lit and electrically shielded room. Participants were seated in a comfortable chair with their eyes 100 cm away from the LCD screen (Asus VZ249HE-W; 23.8¨ screen diagonal). During the experiment, they were presented with the search display of the additional singleton task which was arranged as a 27 × 27 matrix (20° × 13° of visual angle) with a dark grey background (RGB: 60, 60, 60) and a fixation dot in the center. The matrix itself consisted of 458 light-grey (RGB: 134, 134, 134) line elements with a length of 0.7° visual angle, presented either horizontally or vertically. The target stimulus was also a light grey line element, tilted 45° to the right (50% of the trials) or to the left (50% of the trials). The distractor stimulus, on the other hand, was a blue (RGB: 82, 124, 255) or red (RGB: 232, 34, 34) line element, randomly chosen to be horizontal or vertical. Target and distractor stimuli were presented at two out of six fixed locations within the matrix. The vertical midline positions were 4.6° above and below the fixation dot. The lateral positions were 3.8° left and right of the vertical midline and 2.3° above and below the horizontal midline. In half of the experimental trials the target was presented laterally, while the distractor was presented on a vertical midline position. In the other half of the trials the distractor was presented laterally and the target on a vertical midline position. The reward was tied to the color of the distractor (blue or red). For 13 participants the color blue was associated with high rewards and the color red with low rewards. Likewise, for 14 participants the color red was associated with high rewards and the color blue with low rewards. Trials with blue and red distractors were presented equally often. About 27% of trials were target-only trials without a distractor in which the reward magnitude (high or low) was chosen randomly. The task of the participants during the experiment was to respond to the target orientation (tilted to the left or the right) by pressing one of two response buttons on a three-button device with the index finger of their dominant hand (one button for leftward-tilted targets and one button for rightward-tilted targets) while holding a hold-button in between responses. Participants were instructed to react as fast and accurately as they could. While rewards depended on the accuracy of their response, the magnitude of the reward they received (high or low) was determined by the color of the distractor (blue or red).

### 2.4 Procedure

Participants came in for two separate sessions (at least 7 days apart) and performed the additional singleton task while receiving an intravenous infusion of S-ketamine (bolus injection of 10 mg and a maintenance infusion of 0.006 mg/kg/min with a 10% reduction every 10 min) or placebo (0.9% saline solution). This dosage of S-ketamine has already been used in previous studies by our research group (Thiebes et al., 2018; Thiebes et al., 2017). While participants performed the task an EEG was recorded.

At the start of each session, the Positive and Negative Syndrome Scale (PANSS, pre-experiment) was conducted at a desk outside the EEG room. Afterwards, participants were given the opportunity to become familiar with the handling of the button-device and the task at hand. Therefore, first a slowed-down and then a regular block of the task was presented as a training phase. Participants were informed that rewards obtained during this training phase would not be transferred to the main experiment. Thus, every session started with a bank account of zero.

**FIGURE 1:**
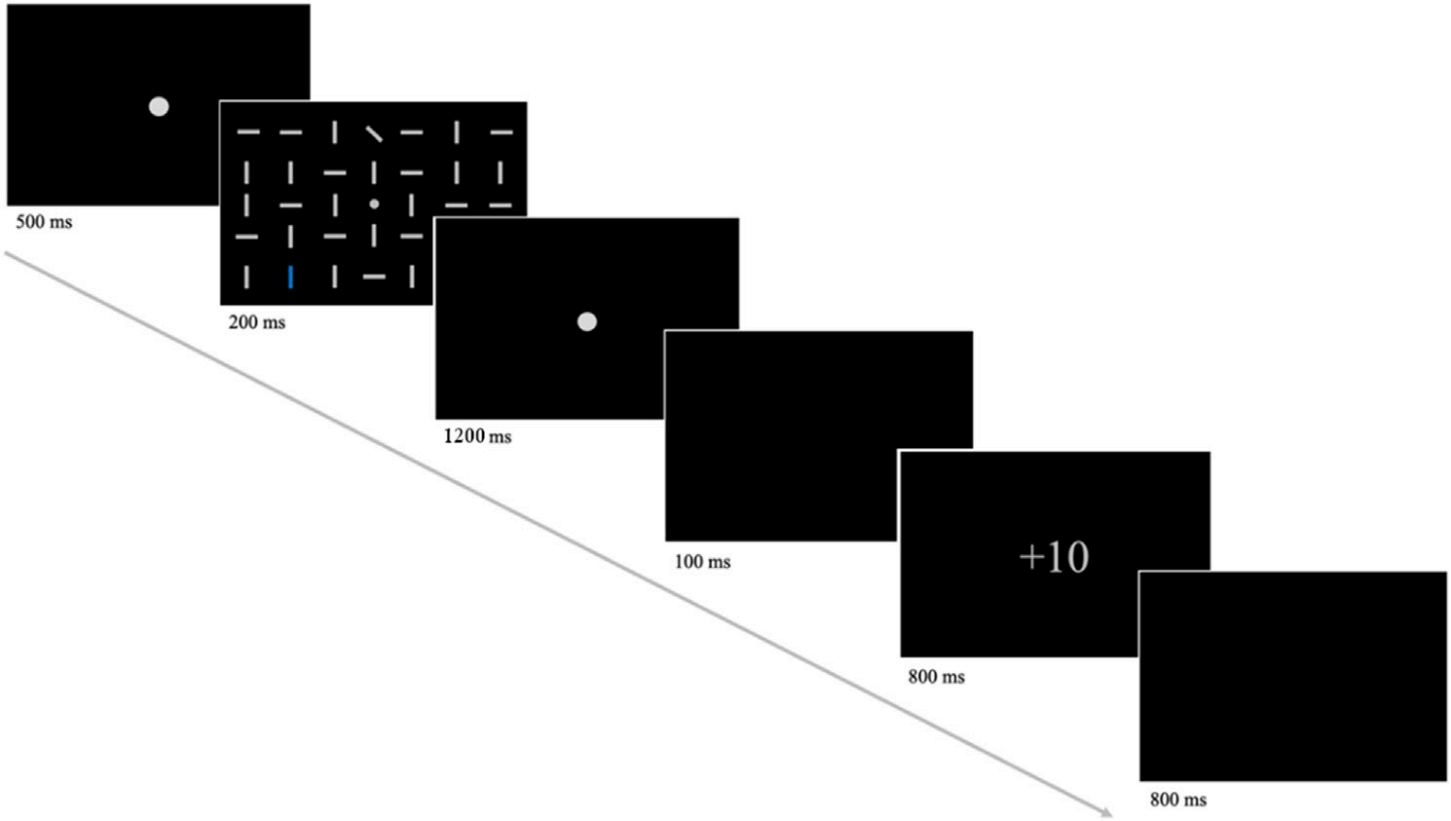
An illustration of the task procedure. First, participants were presented with a fixation dot, which was subsequently replaced by the search display (shown above with a reduced number of line elements). Here, participants were required to respond to the target orientation (leftward-tilted or rightward-tilted) by button press. The assignment of button position (left or right) to target orientation was counterbalanced across participants. Following the response of the participant or a fixation dot for a maximum duration of 1,200 ms, a blank screen and the feedback display were presented. The feedback screen showed +10 for correct responses in high reward trials, +1 for correct responses in low reward trials and +0 for incorrect responses. The amount of reward (+10 or +1) was tied to the color of the distractor (blue or red).

Each trial of the experiment began with the presentation of a central fixation dot for 500 ms, followed by the search display (see Figure 1). After 200 ms the search matrix was replaced by a fixation dot for 1,200 ms or until the response of the participant. Responses slower than 1,400 ms were automatically counted as incorrect. Subsequently, a blank screen was presented for 100 ms, then a feedback display for 800 ms, followed by another blank screen for 800 ms. Participants received 10 points for correct responses in high reward trials and 1 point for correct responses in low reward trials. For incorrect responses, they did not receive any reward. Thus, the feedback display showed +10, +1 or +0. Several factors were counterbalanced across the experiment: distractor/target laterality (lateral position vs. central midline position), distractor color (red vs. blue), target identity (tilted to the left vs. tilted to the right) and actual target/distractor location (position in the matrix). Since the target and distractor positions were chosen to always be on the same side of the horizontal midline, there were 32 possible factor combinations. In each trial one of these combinations was randomly presented to the participant, resulting in 44 trials per block (32 distractor trials and 12 target-only trials). Every participant completed 24 blocks and a total of 1,056 trials per session. After each block participants were shown their averaged response times and accuracy, as well as an account balance, listing their total amount of points and money (in Euros). Because of the ketamine/placebo infusion participants were continuously monitored during the experiment. Heart rate and oxygen saturation were controlled using an ECG and every 10 min a pause between task blocks was used to measure blood pressure. Additionally, after 12 blocks and at the end of the experiment participants were asked about their condition and were given a small questionnaire concerning any symptoms they might experience.

At the end of each session, the PANSS (post-experiment) was conducted. Participants were further monitored until, 1 hour after the experiment, they were picked up by a friend or family member and escorted home.

### 2.5 EEG recording

As with the task and the procedure, the electroencephalographic (EEG) recording was performed similarly to a previous study (Lockhofen et al., 2021). The EEG activity was recorded using Brain Vision Recorder software version 1.21.0303 (Brain Products, Munich, Germany) at a sampling rate of 1,000 Hz with 64 Ag/AgCl electrodes mounted on an elastic cap (ActiCaps, Brain Products, Munich, Germany). Electrodes were arranged according to a modified 10/20 system without electrodes at positions FPz, F9, F10, CP3, CP4, P9, P10, PO7, PO8 and with two additional electrodes at positions PO9 and PO10. Eye movements were recorded with four EOG channels (located on both sides of the outer lid and in the right infra- and supraorbital regions). One electrode at the FCz position was used as a reference, while the electrode at the AFz position was used as ground. The impedances of the electrodes were always kept below 5 kΩ.

### 2.6 Data analysis

#### 2.6.1 PANSS data

To determine if ketamine administration in healthy participants leads to an increase in schizophrenia-like symptoms, total positive and negative symptom scores were calculated for the ketamine session to compare between the pre-experiment and the post-experiment interviews. Since the PANSS is an ordinal scale and all four variables violated the pre-assumption of normal distribution (Shapiro-Wilk, p < 0.001), the data was analyzed nonparametrically using Wilcoxon signed rank tests (corrected for two comparisons).

#### 2.6.2 Behavioral data

Median response times and percent of incorrect responses were calculated for each participant, separately for medication (ketamine or placebo) and reward condition (high or low reward). Afterwards, the data was submitted to a 2 × 2 analysis of variance (ANOVA) with the within-factors medication (ketamine or placebo) and reward condition (high or low reward). To confirm that our distractors lead to longer response times compared to no distractors, the difference between distractor trials and target-only trials was analyzed using Bonferroni-corrected t-tests (corrected for four comparisons).

#### 2.6.3 EEG data processing

EEG data was preprocessed using the EEGLAB toolbox (Delorme and Makeig, 2004). EEG data were down-sampled to 500 Hz and bandpass filtered between 0.5 and 70 Hz for ERP analysis and between 0.5 and 100 Hz for Time-Frequency analysis. The 50 Hz electrical line noise was removed using the Cleanline plug-in of EEGLAB. Artifact-laden channels were identified and removed from the data using the FASTER plug-in within EEGLAB (Nolan et al., 2010). Independent component analysis (ICA) was performed on the continuous EEG data to remove ocular artifacts and generic noise. Artifactual independent components were identified using the IClabel plug-in (Pion-Tonachini et al., 2019) of EEGLAB. EOGs were excluded and the data were segmented into 3-s epochs, starting 1,500 ms before and ending 1,500 ms after stimulus onset. Epochs containing artifacts were removed using a voltage threshold rejection of ±200 μV. After artifact rejection, missing channels were interpolated using spherical interpolation, and the epoched data were re‐referenced to the average of all channels.

#### 2.6.4 Calculation of lateralized ERPs

Similar to our previous work (Lockhofen et al., 2021) we used a systematic lateralization technique (Feldmann-Wüstefeld et al., 2016; Hickey et al., 2009; Woodman and Luck, 2003) to calculate the subcomponents of the N2pc. By subtracting the activity ipsilateral to the distractor from the activity contralateral to the distractor, we calculated the mean lateralized ERPs for all trials with a distractor. To determine the time epochs corresponding to the N2pc subcomponents, we collapsed the mean lateralized ERPs of the electrode pairs PO3/P7 and PO4/PO8 across reward conditions. Each epoch was determined as ±50 ms around the first negative peak in the grand average for trials with lateral targets and central distractors (N_T_), the first negative peak (N_D_) and the first positive peak (P_D_) in the grand average for trials with central targets and lateral distractors. The NT epochs were 175–275 ms for both ketamine and placebo. The N_D_ epochs were 162–262 ms (ketamine) and 163–263 ms (placebo). The P_D_ epochs were 238–338 ms (ketamine) and 228–328 ms (placebo). Afterwards, mean lateralized ERPs were calculated for these epochs, separately for each medication and reward condition. The resulting data was submitted to three 2 × 2 ANOVAs, one for each N2pc subcomponent, with medication (ketamine or placebo) and reward condition (high or low reward) as within-subjects factors. To compare the N_T_ amplitudes and latencies between distractor and target-only trials Bonferroni-corrected t-tests (corrected for four comparisons) were used.

#### 2.6.5 Time-frequency analyses

Time-frequency analysis was performed using the MATLAB scripts provided by (Cohen, 2014). Time-frequency power was computed for the epoch data. It provides a two-dimensional (time × frequency) estimate of changes in spectral power (in dB) relative to the baseline. Time-frequency power was computed by convolving each epoch with complex Morlet wavelets, estimating power across frequencies from 1 to 100 Hz in 100 linearly spaced steps. The wavelet cycles were set at 2 cycles at the lowest frequency (1 Hz), increasing to 7 cycles at the highest frequency (100 Hz). Spectral power was calculated for all channels relative to the −200 to 0 ms prestimulus baseline period. The primary activity of interest was gamma (51–100 Hz) power around the 200–400 ms time window, which overlapped with the time courses of ERP components. Following the previous literature on distractor processing (Kornmayer et al., 2015), gamma power was analyzed at electrodes overlaying occipital scalp regions (O1, Oz, O2).

#### 2.6.6 Statistical analyses of time-frequency power

We performed statistical analysis on gamma (51–100 Hz) power at occipital electrodes (O1, Oz, O2). Total gamma power was extracted from the 200–400 ms time window and averaged across the three occipital electrodes (O1, Oz, O2). A repeated measures ANOVA was performed with medication (placebo, ketamine) and reward (high, low) as factors. The difference between distractor trials and target-only trials was analyzed using Bonferroni-corrected t-tests (corrected for four comparisons).

#### 2.6.7 Additional analyses

In addition to the analyses described above, we performed some additional, exploratory analyses that were not part of our main hypotheses. Specifically, we wanted to assess the relationship between our experimental data (behavioral and EEG data) and the psychopathological symptoms assessed by the PANSS. Since ketamine administration did not lead to a significant increase in positive symptoms (see Section 3.1), we focused our analysis on the negative symptom scale. We performed a median split (MD = 9) and divided participants into a group with subjects who showed increased negative symptoms after ketamine administration and a group with subjects who did not show an increase in negative symptoms after ketamine administration. Using only the data from participants in the high negative symptom group, we calculated bivariate Kendall’s Tau correlations between the PANSS difference scores (post-experiment–pre-experiment) and the response times in the high and low reward conditions. For the EEG data, we calculated bivariate Kendall’s Tau correlations between the PANSS difference scores (post-experiment–pre-experiment) in the high negative symptom group and the N_T_, N_D_ and P_D_ amplitudes/latencies. Finally, we calculated bivariate Kendall’s Tau correlations between the PANSS difference scores (post-experiment–pre-experiment) in the high negative symptom group and total gamma band power in the high and low reward conditions. All resulting values were Bonferroni-corrected.

## 3 Results

### 3.1 PANSS data

**FIGURE 2:**
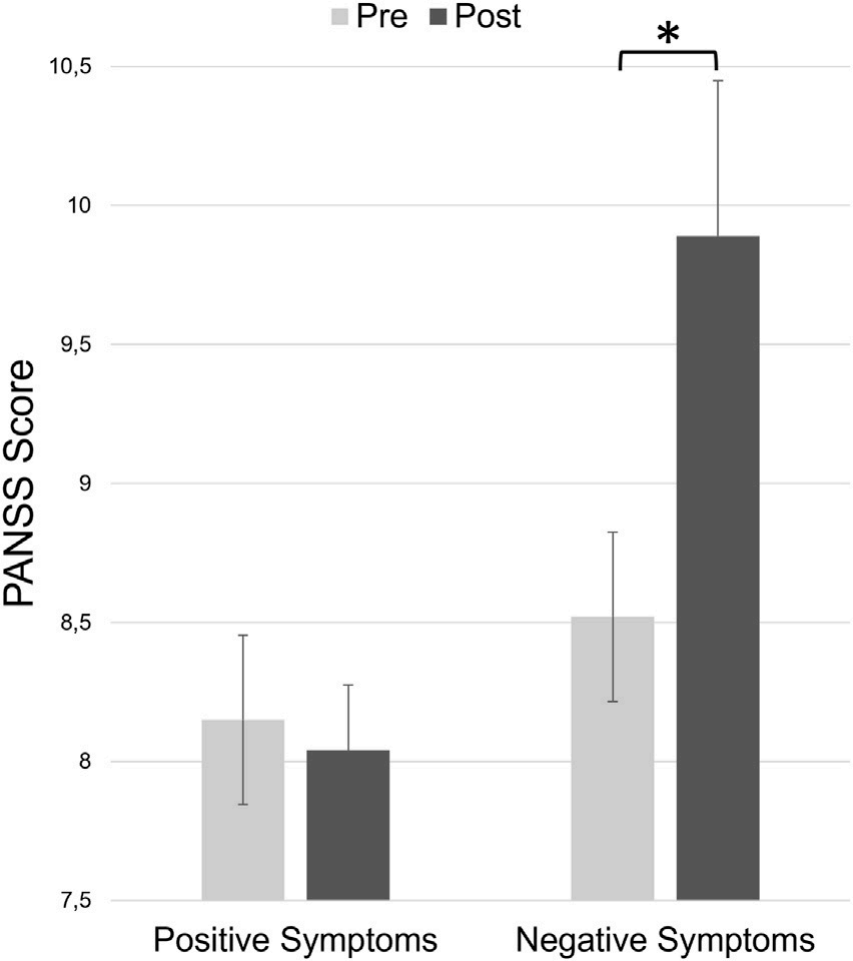
Total positive and negative symptom scores of the PANSS at the beginning of the experiment (before ketamine infusion, pre) and at the end of the experiment (after ketamine infusion, post). Error bars denote the standard error of the mean.

Concerning the negative symptoms, Wilcoxon signed rank tests showed a significant difference between ketamine pre-experiment and ketamine post-experiment (z = 2.499, *p* = 0.024, r = 0.480, see Figure 2). Thus, participants experienced significantly more negative symptoms after ketamine infusion compared to baseline. There was no significant difference in positive symptoms between pre-experiment and post-experiment interviews.

### 3.2 Behavioral data

**FIGURE 3:**
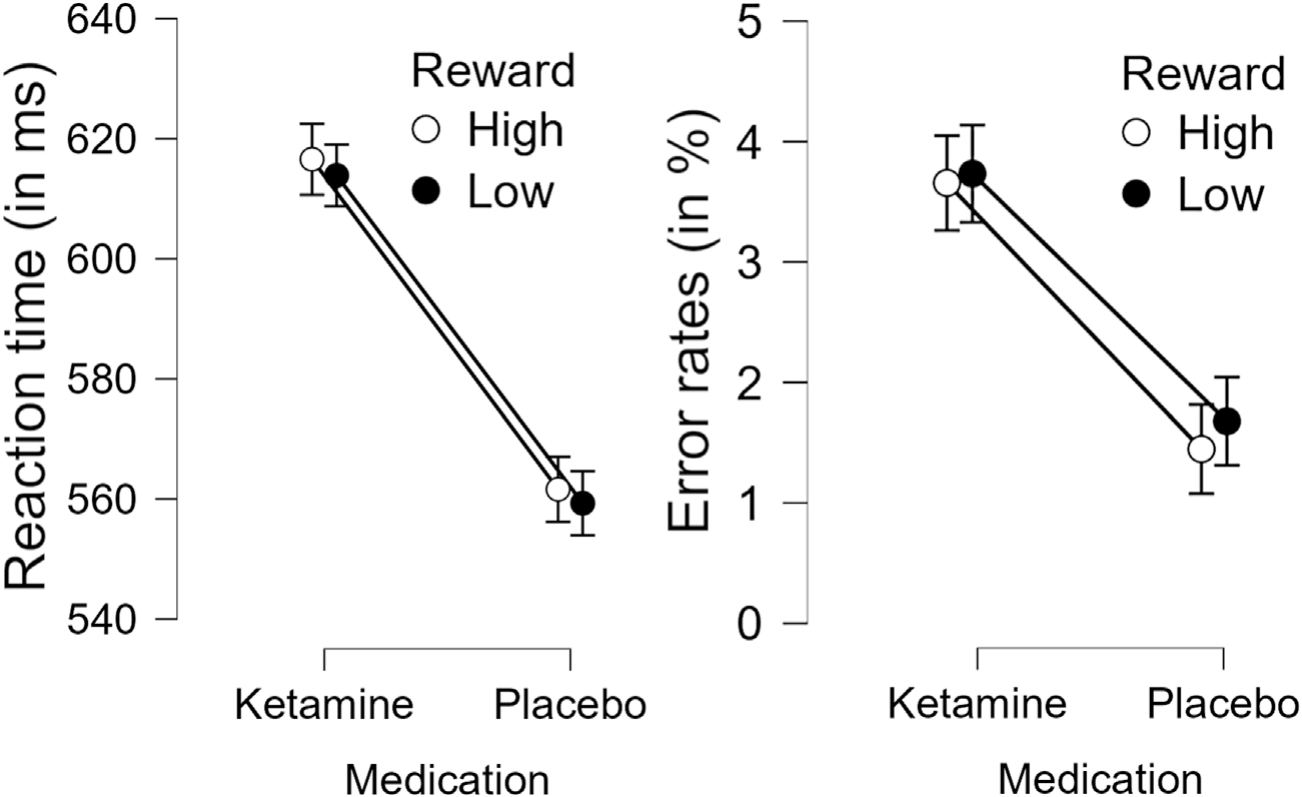
Effect of medication (Ketamine or Placebo) on response times (left) and percent incorrect responses (right) in high and low reward conditions. Error bars denote the standard error of the mean.

Analysis of response accuracy revealed that participants made less than 9% errors in the placebo group and less than 12% errors in the ketamine group. Concerning the response times, the 2 × 2 ANOVA revealed a significant main effect of medication, *F*(1,26) = 35.929, *p* < 0.001, η_p_^2^ = 0.580. This indicates that reaction times were slower under ketamine than under placebo (see Figure 3 (left) and Table 1). The 2 × 2 ANOVA of the percent of incorrect responses also revealed a significant main effect of medication, *F*(1,26) = 11.858, *p* = 0.002, η_p_^2^ = 0.313. Thus, participants made more errors under ketamine than under placebo (see Figure 3 (right) and Table 2). Furthermore, in the ketamine condition, paired t-tests showed a significant difference in response times between target-only trials and high-reward trials, t(26) = 6.966, *p* < 0.001, and between target-only trials and low-reward trials, t(26) = 7.008, *p* < 0.001. In the placebo condition, paired t-tests showed a significant difference in response times between target-only trials and high-reward trials, t(26) = 7.044, *p* < 0.001, and between target-only trials and low-reward trials, t(26) = 9.641, *p* < 0.001. These results show that participants were significantly faster in trials without a distractor than in trials with a distractor (see Table 1).

**TABLE 1:** Descriptive Statistics for ketamine and placebo conditions (response times in ms).

Medication	Ketamine			Placebo		
Reward	HR	LR	TO	HR	LR	TO
N	27	27	27	27	27	27
Mean	616.5	613.8	591.4	561.6	559.2	546.6
Std. Dev.	75.4	65.6	63.86	66.0	63.0	61.8
Max.	844.1	794.9	797.4	761.9	746.0	727.4
Min.	545.1	539.9	530.6	456.4	450.3	449.1

HR, high reward; LR, low reward; TO, target-only; Std. Dev., standard deviation.

**TABLE 2:** Descriptive statistics for ketamine and placebo conditions (percent incorrect responses).

Medication	Ketamine			Placebo		
Reward	HR	LR	TO	HR	LR	TO
N	27	27	27	27	27	27
Mean	3.6	3.7	3.1	1.4	1.6	1.2
Std. Dev.	2.9	3.0	2.7	1.7	1.5	2.3
Max.	11.1	13.8	11.8	8.0	7.0	11.1
Min.	0.0	0.0	0.00	0.0	0.0	0.0

HR, high reward; LR, low reward; TO, target-only; Std. Dev., standard deviation.

### 3.3 ERP data

#### 3.3.1 N_T_ component

Concerning the amplitude of the N_T_, no effect reached significance (p > 0.05). When analyzing the latency of the N_T_, we found a significant medication × reward interaction, *F*(1,26) = 6.343, *p* = 0.018, η_p_^2^ = 0.196. Thus, the reward condition had a differential effect on N_T_ latency under ketamine and placebo (see Tables 3, 4). Post-hoc contrasts revealed that this interaction was driven by a significant difference between high and low reward trials in the placebo condition, t(26) = 3.440, *p* = 0.004, and no significant difference between high and low reward in the ketamine condition. Thus, under placebo, the N_T_ was significantly delayed for high reward compared to low reward trials (see Table 4). In addition, there was also a significant main effect of medication, *F*(1,26) = 22.222, *p* < 0.001, η_p_^2^ = 0.461, as well as a trend towards a significant main effect of reward, *F*(1,26) = 3.170, *p* = 0.087, η_p_^2^ = 0.109.

**TABLE 3:** Descriptive statistics for ERP latency data under ketamine.

Ketamine	N_T_: HR	N_T_: LR	N_T_: TO	N_D_: HR	N_D_: LR	P_D_: HR	P_D_: LR
N	27	27	27	27	27	27	27
Mean Lat. in ms	295.7	294.0	276.0	261.7	274.0	342.1	344.5
Std. Dev.	24.7	24.9	13.1	23.7	21.4	20.9	20.4
Max.	344.0	336.0	298.0	298.0	324.0	378.0	378.0
Min.	262.0	244.0	254.0	210.0	236.0	300.0	296.0

HR, high reward; LR, low reward; TO, target-only; Lat., latency; N_T_, target negativity; N_D_, distractor negativity; P_D_, distractor positivity; Std. Dev., standard deviation.

**TABLE 4:** Descriptive statistics for ERP latency data under placebo.

Ketamine	N_T_: HR	N_T_: LR	N_T_: TO	N_D_: HR	N_D_: LR	P_D_: HR	P_D_: LR
N	27	27	27	27	27	27	27
Mean Lat. in ms	281.8	265.5	273.7	250.0	261.0	333.9	333.7
Std. Dev.	24.8	21.3	14.1	18.1	24.3	25.7	25.6
Max.	318.0	298.0	298.0	284.0	310.0	370.0	378.0
Min.	234.0	210.0	254.0	218.0	220.0	282.0	288.0

HR, high reward; LR, low reward; TO, target-only; Lat., latency; N_T_, target negativity; N_D_, distractor negativity; P_D_, distractor positivity; Std. Dev., standard deviation.

When looking at the effect of the distractor on the N_T_ component, comparing the amplitudes of distractor trials with the amplitudes of target-only trials in the placebo condition revealed significant differences between high reward distractor trials and target-only trials, t(26) = 2.742, *p* = 0.044, and between low reward distractor trials and target-only trials, t(26) = 2.753, *p* = 0.044. Under ketamine, the same comparisons revealed trends towards significant differences between high reward distractor trials and target-only trials, t(26) = 2.563, *p* = 0.068, and between low reward distractor trials and target-only trials, t(26) = 2.538, *p* = 0.068. Therefore, the amplitudes of the N_T_ component were larger for target-only trials than for distractor trials, especially under placebo (see Tables 5, 6). Concerning the latencies, comparing distractor trials with target-only trials in the ketamine condition revealed significant differences between high reward distractor trials and target-only trials, t(26) = 3.704, *p* = 0.004, and between low reward distractor trials and target-only trials, t(26) = 3.186, *p* = 0.016. Under placebo, the same comparisons did not reach significance. Thus, under ketamine the N_T_ component was significantly delayed in distractor trials compared with target-only trials (see Tables 3, 4).

**TABLE 5:** Descriptive statistics for ERP amplitude data under ketamine.

Ketamine	N_T_: HR	N_T_: LR	N_T_: TO	N_D_: HR	N_D_: LR	P_D_: HR	P_D_: LR
N	27	27	27	27	27	27	27
Mean Amp. in µV	−1.0	−1.1	−2.8	−0.4	−0.1	0.1	0.3
Std. Dev.	0.7	0.8	3.6	0.6	0.5	0.6	0.7
Max.	0.5	0.2	8.0	0.7	1.2	1.4	2.7
Min.	−3.1	−3.5	−8.6	−1.9	−1.4	−1.1	−1.3

HR, high reward; LR, low reward; Amp., amplitude; TO, target-only; N_T_, target negativity; N_D_, distractor negativity; P_D_, distractor positivity; Std. Dev., standard deviation.

**TABLE 6:** Descriptive statistics for ERP amplitude data under placebo.

Ketamine	N_T_: HR	N_T_: LR	N_T_: TO	N_D_: HR	N_D_: LR	P_D_: HR	P_D_: LR
N	27	27	27	27	27	27	27
Mean Amp. in µV	−1.0	−1.0	−2.4	−0.3	−0.2	0.0	−0.0
Std. Dev.	0.7	0.6	2.86	0.4	0.5	0.7	0.6
Max.	0.1	0.1	4.6	0.3	0.6	1.5	0.9
Min.	−2.9	−2.7	−9.5	−1.2	−1.5	−1.7	−1.8

HR, high reward; LR, low reward; TO, target-only; Amp., amplitude; N_T_, target negativity; N_D_, distractor negativity; P_D_, distractor positivity; Std. Dev., standard deviation.

#### 3.3.2 N_D_ component

**FIGURE 4:**
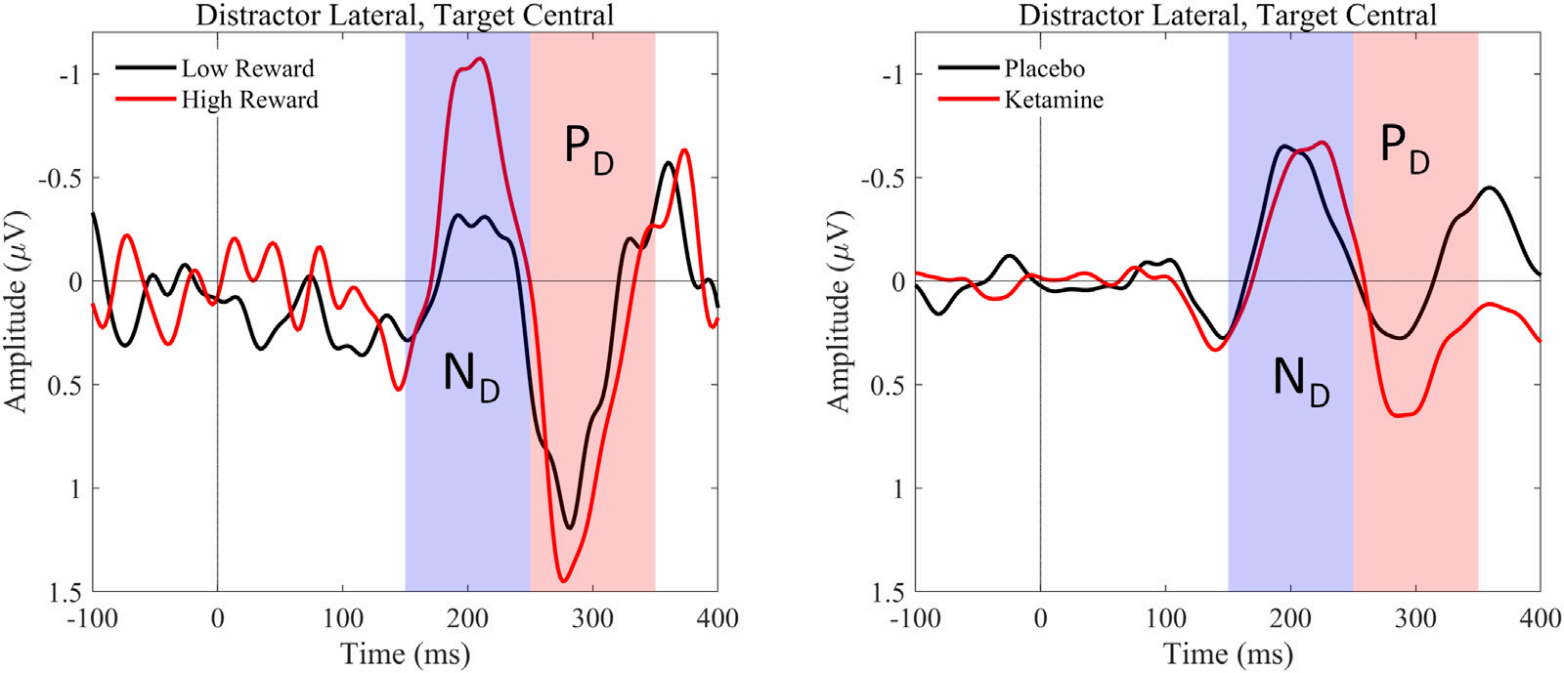
Graphical representation of the grand average ERPs recorded for centrally presented targets and laterally presented distractors. ERPs were pooled over the electrode pairs PO3/P7 and PO4/P8. Afterwards, we calculated the difference waves contralateral–ipsilateral to distractor position. The left graphic shows the lateralized ERPs evoked by high and low rewards, averaged over medication conditions. The waves elicited by high-reward distractors are presented in red, the waves elicited by low-reward distractors in blue. The right graphic shows the lateralized ERPs evoked by ketamine and placebo, averaged over reward conditions. The waves elicited by distractors under ketamine are presented in red, the waves elicited by distractors under placebo in blue. The peaks of the N2pc subcomponents, distractor negativity (N_D_) and distractor positivity (P_D_), are marked.

With regard to the N_D_ amplitude, we found a significant main effect of reward, *F*(1,26) = 5.086, *p* = 0.033, η_p_^2^ = 0.164. Thus, participants had significantly larger mean N_D_ amplitudes in high reward than in low reward trials (see Figure 4 (left) and Tables 5, 6). When looking at the latency of the N_D_, we found a significant main effect of medication, *F*(1,26) = 13.338, *p* = 0.001, η_p_^2^ = 0.339, as well as a significant main effect of reward, *F*(1,26) = 9.938, *p* = 0.004, η_p_^2^ = 0.277. Thus, the mean N_D_ peak was later under ketamine than under placebo (see Figure 4 (right) and Tables 3, 4) and for low compared to high reward trials (see Figure 4 (left) and Tables 3, 4).

#### 3.3.3 P_D_ component

Concerning the P_D_ amplitude, we found a significant main effect of medication, *F*(1,26) = 4.979, *p* = 0.034, η_p_^2^ = 0.161. Thus, participants had significantly larger mean P_D_ amplitude under ketamine than under placebo (see Figure 4 (right) and Tables 5, 6). With regard to the P_D_ latency, we also found a significant main effect of medication, *F*(1,26) = 6.034, *p* = 0.021, η_p_^2^ = 0.188. Therefore, the mean P_D_ peak was later under ketamine than under placebo (see Figure 4 (right) and Tables 3, 4). No other effects reached significance.

### 3.4 Time frequency data

#### 3.4.1 Gamma power

**FIGURE 5:**
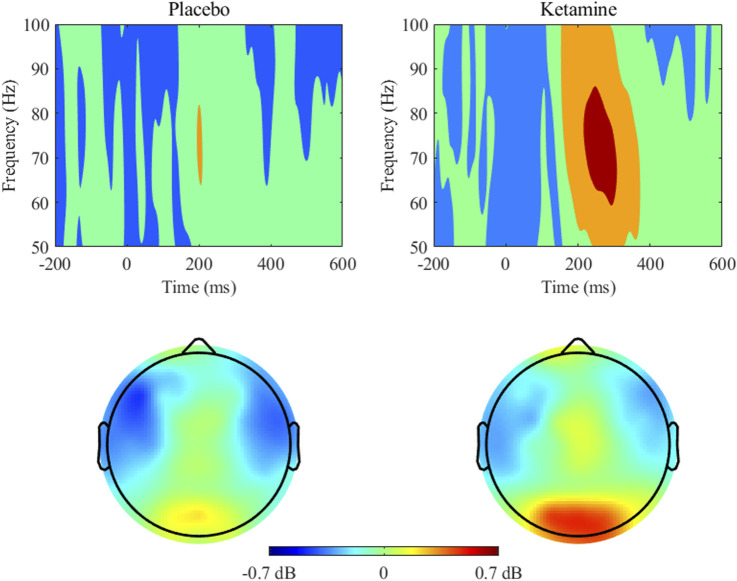
Gamma (51–100 Hz) power and topographic maps for placebo and ketamine conditions. Time 0 indicates the stimulus onset. Power (dB) values are averaged across occipital electrodes (O1, Oz, O2). Topographic maps show power distribution around 200–400 ms time window at the 51–100 Hz band.

The 2 × 2 ANOVA of occipital gamma power revealed a significant main effect of medication, *F*(1,26) = 8.172, *p* = 0.008, η_p_^2^ = 0.239. Thus, participants showed higher total gamma power in the occipital regions under ketamine compared to the placebo (see Figure 5, Table 7).

**TABLE 7:** Descriptive statistics for total occipital gamma power under ketamine and placebo.

Medication	Ketamine			Placebo		
Reward	HR	LR	TO	HR	LR	TO
N	27	27	27	27	27	27
Mean	0.5	0.5	0.4	0.1	0.2	0.1
Std. Dev.	0.7	0.7	0.8	0.2	0.3	0.2
Max.	3.0	3.1	3.2	0.6	0.7	0.7
Min.	−0.1	−0.3	−0.4	−0.1	−0.4	−0.3

HR, high reward; LR, low reward; TO, target-only; Std. Dev., standard deviation.

**FIGURE 6:**
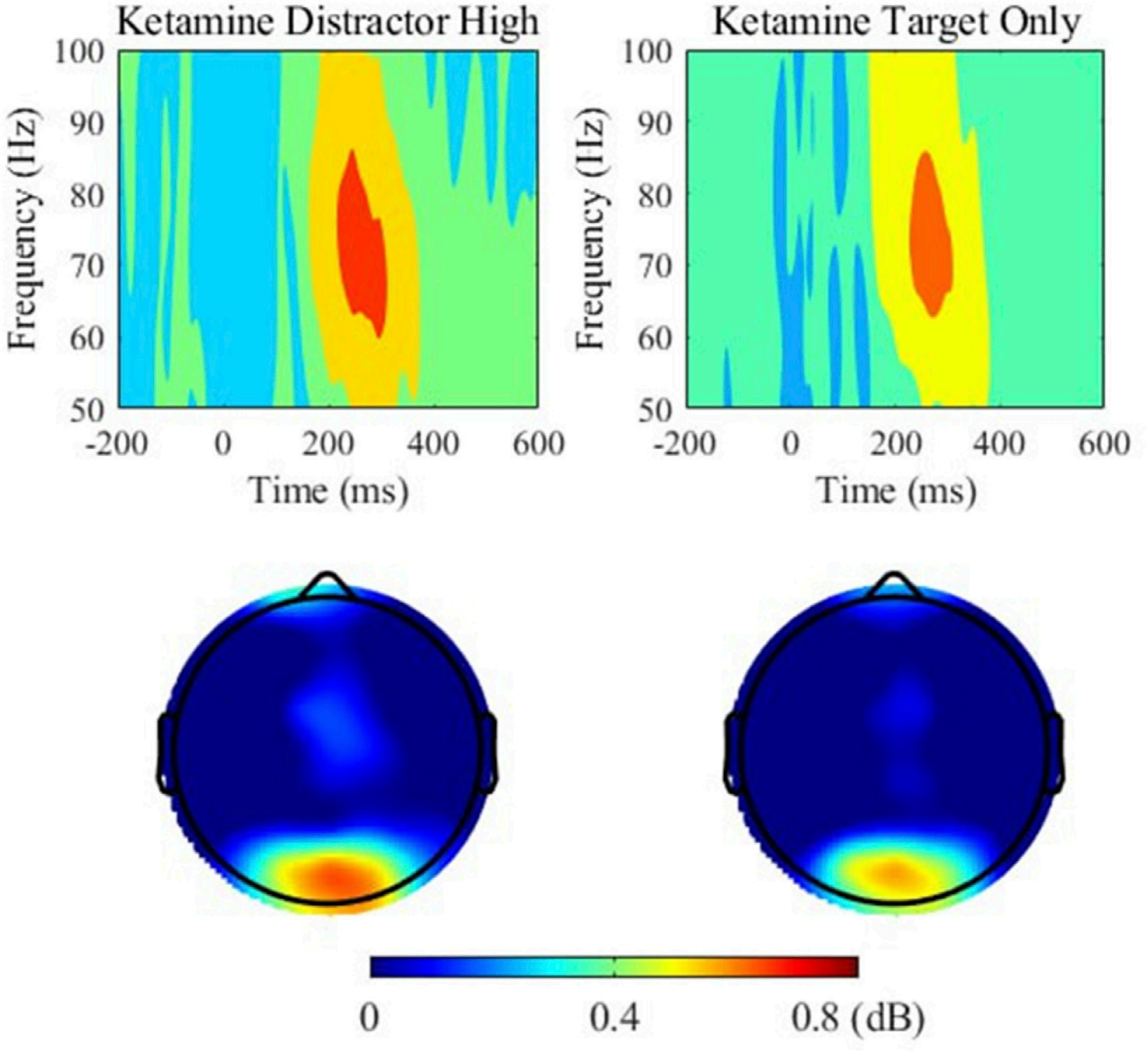
Gamma (51–100 Hz) power and topographic maps for high-rewarded distractor trials (left) and target only trials (right) under ketamine. Time 0 indicates the onset of the stimulus. Power (dB) values are shown as averaged across electrodes overlaying occipital regions (O1, Oz, O2). Topographic maps show the distribution of power around 200–400 ms time window at the 51–100 Hz band.

Furthermore, paired t-tests showed a significant difference in gamma power between target-only trials and high-reward trials, t(26) = 2.923, *p* = 0.028 and a trend towards a significant difference between target-only trials and low-reward trials, t(26) = 2.602, *p* = 0.060. This demonstrates that under ketamine, gamma power was significantly higher in distractor trials than in target-only trials, especially when distractors were associated with high rewards (see Figure 6). There were no significant differences in gamma power between distractor and target-only trials in the placebo condition.

### 3.5 Additional analyses

**FIGURE 7:**
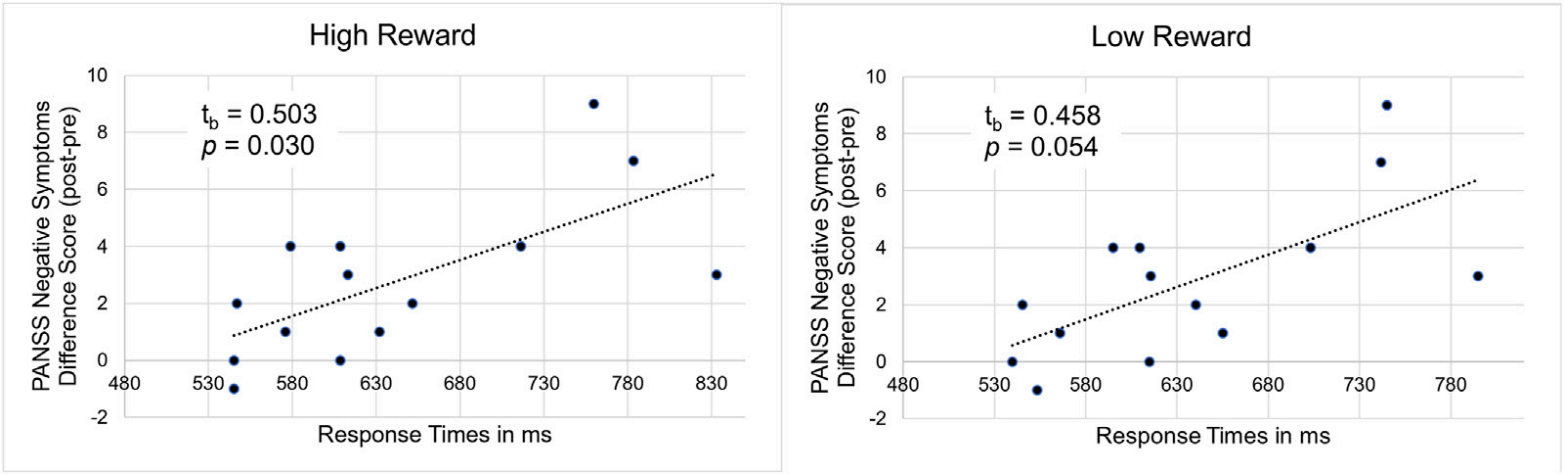
Illustration of the correlation between PANSS negative symptom difference scores (post-experiment–pre-experiment) and response times under ketamine in high (left) and low (right) reward conditions.

Concerning the behavioral data, we found a significant correlation between the post–pre-experiment difference in the PANSS negative symptoms score and the response times in the high reward condition as well as a trend towards a significant correlation in the low reward condition, t_b_ = 0.503 (95%CI 0.143–0.746), *p* = 0.030 (high reward, see Figure 7 (left)) and t_b_ = 0.458 (95%CI 0.084–0.718), *p* = 0.054 (low reward, see Figure 7 (right)). This shows that for participants with a high number of negative symptoms in the post-experiment interview an increase in negative symptoms after ketamine infusion compared to baseline was accompanied by longer response times.

**FIGURE 8:**
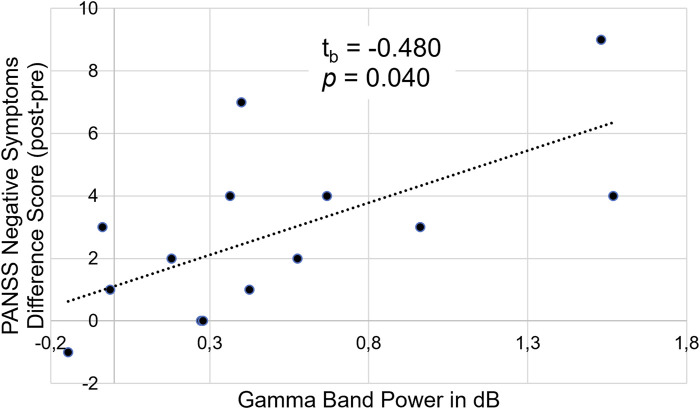
Illustration of the correlation between PANSS negative symptom difference scores (post-experiment–pre-experiment) and gamma band power (in dB) in the high reward condition.

Concerning the EEG data, we found no correlations between any ERP amplitudes or latencies with the PANSS difference scores. However, there was a significant correlation between the PANSS difference scores and gamma band power in the high reward condition, t_b_ = 0.480 (95%CI 0.113–0.732), *p* = 0.040. This shows that for participants with a high number of negative symptoms post-experiment an increase in negative symptoms after ketamine infusion compared to baseline was related to a stronger gamma band response (see Figure 8).

## 4 Discussion

### 4.1 Summary of results

In recent years, the glutamate hypothesis of schizophrenia has become increasingly relevant. It was shown that ketamine elicits cognitive deficits and psychotic symptoms in healthy subjects, resembling those witnessed in patients with schizophrenia. The present study confirms these results by showing that ketamine leads to increased response times and error rates in a visual attention task with rewarded distractors, which is in line with previous results found in schizophrenic patients (Lesh et al., 2011; Carter et al., 2010; Carter et al., 2013). However, our study expands these results by demonstrating that healthy subjects under ketamine show aberrant distractor processing, as well as increased occipital gamma-band activity compared to placebo.

### 4.2 PANSS results

Concerning the psychopathological effects elicited by ketamine administration in healthy volunteers, we found that participants experienced significantly more negative symptoms after ketamine infusion compared to baseline. Since it has been demonstrated that ketamine can induce transient schizophrenia-like symptoms in healthy individuals (Beck et al., 2020; Pennybaker et al., 2017), this result was to be expected. It also matches the findings of other studies that used the PANSS to assess psychopathology after ketamine administration (Curic et al., 2019; Curic et al., 2021; Carter et al., 2010). Furthermore, it reinforces the assumption that the emergence of negative symptoms in schizophrenic patients might be related to the glutamatergic system (Umbricht et al., 2014). This could explain why dopaminergic drugs sometimes fail to alleviate negative and cognitive symptoms (Greener, 2024; McCutcheon et al., 2020).

### 4.3 Behavioral and ERP results

#### 4.3.1 Effects of medication

After ketamine administration, participants showed generally longer response times and increased error rates in the visual attention task, which supports the assumption that acute ketamine administration in healthy participants impairs cognition (Zhornitsky et al., 2022) and can induce attentional deficits similar to those found in schizophrenic patients (Oranje et al., 2000; Umbricht et al., 2002). In the EEG we found that ketamine had a strong effect on the distractor-related subcomponents of the N2pc. Ketamine delayed attentional capture by salient distractors as well as active suppression of these distractors. This is in line with the assumption of abnormal salience processing in patients with schizophrenia (Kornmayer et al., 2018; Kornmayer et al., 2015).

Previous studies have already shown that ketamine affects distractor-related ERP parameters, using variants of an oddball task and the P3a and P3b EEG components as measures of stimulus-driven and task-related attentional processing (Rosburg and Schmidt, 2018; Schwertner et al., 2018). Yet, the authors found that these attentional effects were not always accompanied by differences in task performance, except for studies with high working memory load ((Rosburg and Schmidt, 2018), but see (Oranje et al., 2000; Watson et al., 2009; Gunduz-Bruc et al., 2012)). In our study we found pronounced deficits in response times as well as increased error rates under ketamine compared to placebo. The reason for this discrepancy might be that in our study participants not only had to respond to targets and suppress salient distractors, but they were also presented with a full screen of non-salient distractors. Moreover, the salient distractors were associated with differing rewards, dependent on the distractor color. Learning these non-instructed reward-contingencies might have increased cognitive load, relative to simple target detection tasks.

In addition to a delay in distractor processing, participants in our study showed an increased P_D_ amplitude under ketamine compared to placebo. The distractor positivity is assumed to be an index of distractor suppression (Hickey et al., 2009; Drisdelle and Eimer, 2021), that overrides attentional capture and facilitates attention to targets (Sawaki and Luck, 2013; Sawaki and Luck, 2011). It indicates not only that a stimulus is not attended to, but that it is actively suppressed (Gaspar and McDonald, 2014; Sawaki and Luck, 2010). Previous studies have shown that ketamine affects top-down attentional control, especially when distractor suppression is required (Fuchs et al., 2015). However, the results for patients with schizophrenia were mixed. By using an attentional task with target colors and matching or non-matching distractors, it was shown that healthy subjects were able to suppress target-color distractors, indicated by an increased P_D_ component, while patients with schizophrenia showed hyperfocusing on target-color distractors and no active suppression (Sawaki et al., 2017). At the same time, several studies have demonstrated that patients with schizophrenia do not show a general deficit in the ability to suppress distracting information (Luck et al., 2014; Gold et al., 2009; Westerhausen et al., 2013).

In our study the increased distractor suppression was not accompanied by an increase in attentional capture or target prioritization, as indexed by no significant change in N_T_/N_D_ amplitude in the ketamine condition compared to placebo. Yet, we did find slower reaction times and increased error rates in response to targets under ketamine. These changes cannot be due to a general slowing of response times under ketamine, since participants in both conditions were significantly faster when no distractor was presented (target-only trials). Therefore, we can only assume that participants in the ketamine condition show a failed compensation by engaging in more active suppression of salient, reward-related distractors, which did not affect performance in the attentional task.

Target processing, reflected in the N_T_ component of the N2pc, was also affected by ketamine administration, but not as much as the distractor processing, which is in line with earlier results that found no difference in attentional allocation to visual targets (Luck et al., 2006).

In summary, blocking the NMDAR with ketamine in a sample of healthy participants leads to deficits in a visual attention task and to an abnormal suppression of salient distractors with little to no effect on target processing. Future studies should focus on schizophrenic patients to clarify whether this abnormal processing of physically salient stimuli might be involved in the pathophysiological genesis of schizophrenic symptoms.

#### 4.3.2 Effects of reward magnitude

Contrary to our expectations, we did not find an increase in response times for high reward compared to low reward trials. This missing effect of reward magnitude matches the results from a previous study in which we could not find a significant response time difference between high and low rewards in the distractor reward group (DR), only in the target reward group (TR, (Lockhofen et al., 2021)). One reason for the missing response time difference between high and low reward distractors might be that participants were not previously informed about the details of the reward scheme. Previous research has shown that participants learn reward contingencies over time (Feldmann-Wüstefeld et al., 2016; Failing and Theeuwes, 2017). However, awareness of the distractor-reward relationship was found to be crucial, especially when the search display is heterogeneous (Failing and Theeuwes, 2017). On the other hand, reward effects can also be present in participants who are naive towards the reward scheme (Feldmann-Wüstefeld et al., 2016).

Comparing distractor-trials with target-only trials demonstrated that participants under ketamine as well as under placebo were significantly faster in trials without a distractor. This was expected and indicates that our reward-related distractors had a detrimental effect on attentional processing, irrespective of reward magnitude or medication.

While we did not find an effect of reward on response times and error rates, we found a significantly higher N_D_ component in the EEG for high compared to low reward trials, which demonstrates that high-reward distractors had a stronger attentional capture effect than low-reward distractors. This stronger attentional capture effect for distractors associated with high rewards was expected and is in line with previous results (Feldmann-Wüstefeld et al., 2016). It shows that in our study high-reward distractors did in fact capture attention more than low-reward distractors, even in the absence of behavioral effects. This dissociation (strong ERP effects, weak behavioral effects) might be due to task difficulty. In our experiment participants solved the task with high accuracy, even in the ketamine condition. Therefore, we can assume that the task was relatively easy for them. That is why any differences occurring on the neurophysiological level might not have translated into behavioral effects.

In addition, we found an interaction between reward magnitude and medication on the latency of target prioritization, reflected in the N_T_ component of the N2pc. Under placebo, the latency of the N_T_ was delayed for high reward distractors compared to low reward distractors, which supports our assumption that reward-related distractors would impair target processing (Feldmann-Wüstefeld et al., 2016). This reward-dependent effect was absent under ketamine, indicating that NMDAR antagonists can compromise reward processing (Chow and Beckmann, 2018; Fitzpatrick and Morrow, 2017; Francois et al., 2016). In contrast to previous studies (Lockhofen et al., 2021; Feldmann-Wüstefeld et al., 2016), we did not find an effect of reward magnitude on the amplitude of the N_T_ component. We also did not find any other interactions between medication and reward magnitude.

To summarize, our EEG results indicate that high reward distractors capture attention more than low reward distractors. However, the effects of ketamine administration were mostly unaffected by reward magnitude, which leads us to assume that glutamatergic modulation mainly affects early attention and salience processing with almost no effect on reward processing.

### 4.4 Gamma band results

In the present study, we found increased occipital total gamma power under ketamine compared to placebo. Several studies have reported abnormal gamma band oscillations under ketamine (Nottage et al., 2023; Curic et al., 2021; Grent-‘t-Jong et al., 2018; Shaw et al., 2015). Specifically, increases in spontaneous and resting-state gamma power were reported after NMDAR antagonist administration in pre-clinical samples as well as in healthy participants (Hirano and Uhlhaas, 2021). However, some studies also found an increase in task-related gamma power following ketamine administration (Grent-‘t-Jong et al., 2018; Shaw et al., 2015).

In addition to a general increase in gamma band power under ketamine, we also found increased gamma band power in salient distractor trials compared to target-only trials. We expected this result based our previous findings (Kornmayer et al., 2015), in which we demonstrate a significantly increased early evoked gamma band response for salient distractors. Nevertheless, it has to be pointed out that in the present study we report total gamma band power in a time range of 250–350 ms after stimulus presentation, not evoked gamma 50–150 ms after stimulus onset. Concerning the effect of reward, reward modulation did not increase or decrease gamma power in our experiment. This in agreement with previous studies demonstrating that reward processing is mainly related to beta and theta band oscillations (Andreou et al., 2017; Leicht et al., 2020). Thus, our results indicate that gamma oscillations induced by ketamine administration in healthy participants are more involved in attention and early salience processing than in reward processing.

Disturbances of gamma band oscillations after NMDAR blockade by ketamine are believed to be due to a modulation of excitatory input from pyramidal cells on fast-spiking parvalbumin interneurons (Carlén et al., 2012) which are prominently involved in the generation of gamma band oscillations (Sohal et al., 2009). Since gamma band oscillations were found to be correlated with performance in cognitive tasks, it was assumed that they might underlie the cognitive disturbances in patients with psychiatric illnesses, such as schizophrenia (Uhlhaas, 2013; Roopun et al., 2008; Sun et al., 2011). Indeed, schizophrenic patients do show aberrant gamma band oscillations (Leicht et al., 2010; Andreou et al., 2015; Kornmayer et al., 2018; Uhlhaas et al., 2008; Cho et al., 2006; An et al., 2024). However, at first glance the directions of gamma band power abnormalities in patients and healthy subjects under ketamine seem to be inconsistent. While we find increased gamma band power associated with negative symptoms in healthy participants after ketamine administration, other studies from our group found a reduced gamma band response for patients with schizophrenia (Leicht et al., 2010; Leicht et al., 2015) as well as for healthy subjects under ketamine (Curic et al., 2019). At the same time, further results from our group demonstrate that patients with schizophrenia and subjects with schizotypal personality disorder exhibit an increased gamma band response in association with their clinical symptoms (Kornmayer et al., 2018; Kornmayer et al., 2015). Therefore, we propose that a more differentiated view is needed. When taking into account the nature of the cognitive tasks that the subjects had to perform, one has to realize that the results within a task domain (auditory or visual) are in fact consistent. Studies that report results from auditory tasks (Leicht et al., 2010; Curic et al., 2019; Leicht et al., 2015) generally find a reduction in early gamma band power for schizophrenic patients and healthy participants under ketamine. On the other hand, studies that report results from visual tasks (Kornmayer et al., 2018; Kornmayer et al., 2015) find an increase in gamma band power in response to salient visual stimuli. Thus, the findings from the present study are consistent with our previous works by demonstrating increased gamma band power in response to salient distractors within a visual attention task.

### 4.5 Relation to clinical symptoms

In our additional, exploratory analyses we found that for subjects responding to ketamine treatment (showing increased negative symptoms) reaction times were correlated with the number of negative symptoms (post-experiment–pre-experiment). This indicates that the attentional impairments found in the behavioral data can be mainly attributed to a ketamine-induced increase in negative symptoms and to a lesser extent to an increase in positive symptoms. This is in line with previous studies (Thiebes et al., 2017; Honey et al., 2008; Mueller et al., 2018) and might be attributed to impairments in prefrontal function (Silver and Feldman, 2005). Additionally, we could demonstrate that the increase in gamma band power under ketamine was associated with an increase in negative symptoms, which is consistent with previous studies showing an association between the gamma band response, NMDAR antagonism and negative symptoms in patients with schizophrenia (Curic et al., 2019; Leicht et al., 2015; Hong et al., 2010).

### 4.6 Limitations

This study is not without limitations. One limitation concerns the lack of blinding to treatment. While participants were not explicitly told what medication they would receive, most of them could infer based on their physical reaction and the symptoms they experienced. While previous studies (Beck et al., 2020) have shown that the blinding status did not influence the effect size for positive or negative symptoms, functional unblinding is a problem of many ketamine studies. The effects of functional unblinding are highly dependent on the beliefs of the participant. If the participants expect a positive effect of ketamine, they might become more focused. Conversely, if the participants expect negative effects, they might become more anxious or distracted. Using another psychotomimetic drug instead of NACl as a control condition could reduce this bias. However, other psychoactive substances, such as benzodiazepines, also show neurophysiological effects, which might make them unsuitable as active control substances in our study. Another limitation concerns the use of the median split to differentiate between participants who responded to ketamine treatment and experienced an increase in negative symptoms and participants who did not. It is possible that using this procedure could have led to a loss of information, a loss of power and to an increase in Type 1 errors. At the same time, there is evidence from simulation studies that these effects are in most cases negligible (Iacobucci et al., 2015).

### 4.7 Conclusions

This study is the first to investigate the interaction of attention and reward processing under ketamine compared to placebo. Ketamine administration in healthy participants mainly affected attentional processes associated with the salient distractor and showed little or no effect on target or reward-related processes. Therefore, this study supports the assumption that modulation of glutamatergic signaling affects early salience processing. In addition, the findings from the present study are consistent with our previous work (Kornmayer et al., 2015) by demonstrating increased gamma band power in response to salient distractors within a visual attention task, as well as a correlation between gamma band power and clinical symptoms induced by ketamine administration. Overall, this study further emphasizes the role of the glutamate system in developing dysfunctional gamma band oscillations, early salience processing aberrations and negative symptoms in patients with schizophrenia.

## Data Availability

The datasets presented in this article are not readily available because of restrictions of the ethics committee. Requests to access the datasets should be directed to the corresponding author.
